# A 7‐mm Covered TIPS Reduces Hepatic Encephalopathy Without Increasing Rebleeding in Cirrhotic Patients With Small Liver: A Randomized Study

**DOI:** 10.1111/liv.70875

**Published:** 2026-09-10

**Authors:** Fuquan Liu, Kan Zhang, Yu Zhang, Quanwei He, Mingming Meng, Xuemei Ma, Bing Zhu, Yifan Wu, Linjing An, Yifan Lv, Fuhuang Lin, Ke Zhang, Bowen Liu, Xiujuan Chang, Huiguo Ding, Jiangtao Liu, Yan Chen, Yongping Yang

**Affiliations:** ^1^ Liver Disease Center Hainan Hospital of Chinese PLA General Hospital Sanya China; ^2^ Interventional Therapy The Fifth Medical of Chinese PLA General Hospital Beijing China; ^3^ Pulmonary and Critical Medicine The 8th Medical Center of Chinese PLA General Hospital Beijing China; ^4^ Department of Gastroenterology and Interventional Therapy, Beijing Shijitan Hospital Capital Medical University Beijing China; ^5^ Department of Interventional Therapy Hainan Provincial People's Hospital Haikou China; ^6^ Department of General Surgery, Beijing Ditan Hospital Capital Medical University Beijing China; ^7^ Center for Hepatology and Gastroenterology, Beijing You‐An Hospital Capital Medical University Beijing China

**Keywords:** hepatic encephalopathy, liver cirrhosis, stent diameter, transjugular intrahepatic portosystemic shunt, variceal rebleeding

## Abstract

**Background/Aims:**

International guidelines recommend initiating transjugular intrahepatic portosystemic shunt (TIPS) placement with an 8‐mm stent. However, there is an evident lack of randomized controlled trials evaluating TIPS diameters < 8 mm in cirrhotic patients with a relatively small liver. The aim of this study was to determine whether 7 mm‐covered TIPS, compared with 8‐mm stents, could achieve comparable shunt function with a lower incidence of hepatic encephalopathy (HE).

**Methods:**

In this multicenter randomized controlled trial, patients with cirrhosis and relatively small liver were randomized 1:1 to receive TIPS with a 7‐mm (*n* = 92) or 8‐mm (*n* = 92) covered stent to prevent variceal rebleeding. The primary endpoint was the incidence of overt HE after randomization. All‐cause rebleeding, orthotopic liver transplantation (OLT)‐free survival and a composite of these outcomes, were designated as secondary endpoints.

**Results:**

Among the 184 enrolled patients, the predominant etiologies of liver cirrhosis were hepatitis B virus infection (56.0%) and alcohol‐related liver disease (20.7%). Over a median follow‐up of 26.5 months, overt HE occurred in 19 patients (20.7%) in the 7‐mm group and 33 patients (35.9%) in the 8‐mm group. The 2‐year cumulative incidence of overt HE was significantly lower in the 7‐mm group than in the 8‐mm group (21.4% vs. 37.2%, *p* = 0.02). Stent diameter, post‐TIPS portosystemic pressure gradient, pre‐covert HE and MELD‐Na score were identified as independent risk factors for overt HE. The rates of shunt dysfunction were statistically similar between groups (8.7% vs. 8.7%, *p* = 1.0), as were 2‐year rebleeding rates (10.9% vs. 9.8%, *p* = 0.81) and OLT‐free survival rates (91.3% vs. 88.0%, *p* = 0.82).

**Conclusions:**

A 7‐mm covered TIPS demonstrated comparable shunt function to an 8‐mm covered stents, with a significantly lower risk of overt HE. These findings support consideration of 7‐mm TIPS stents for preventing variceal rebleeding in cirrhotic patients with a small liver who are undergoing TIPS.

**Trail Registration:**

ClinicalTrials.gov, NCT02541825

## Highlights

- In patients with cirrhosis and a relatively small liver, initiating a transjugular intrahepatic portosystemic shunt (TIPS) with an 8‐mm covered stent does not reduce hepatic encephalopathy (HE) and may increase hepatic insufficiency. The optimal stent diameter for preventing variceal rebleeding by TIPS remains uncertain.
- We therefor conducted this study and found that TIPS with 7‐mm covered stents was associated with a significantly lower risk of overt HE, while maintaining similar rebleeding and survival rates compared with 8‐mm stents. These findings suggest that the use of 7‐mm stents should be considered for the prevention of variceal rebleeding in cirrhotic patients with a relatively small liver who are undergoing TIPS.

## Lay Summary

In patients with cirrhosis and a relatively small liver being considered for an 8‐mm TIPS procedure, the hepatic encephalopathy does not reduce and may increase hepatic insufficiency. Our findings suggest that the use of 7‐mm stents should be considered for the prevention of variceal rebleeding in cirrhotic patients with a relatively small liver who are undergoing TIPS.

**Abbreviations**
CI: confidence interval
HR: hazard ratio
IQR: interquartile range
MELD: model for end‐stage liver disease
OHE: overt hepatic encephalopathy
PPG: portosystemic pressure gradient
RCT: randomized controlled trial
TIPS: transjugular intrahepatic portosystemic shunt

## 1 Introduction

Variceal bleeding represents a critical complication of portal hypertension and is a leading cause of mortality among patients with cirrhosis [1, 2]. Since its introduction into clinical practice, transjugular intrahepatic portosystemic shunt (TIPS) has been recommended for patients with cirrhosis experiencing uncontrolled acute variceal haemorrhage during endoscopy or for those who experience rebleeding post‐endoscopic variceal ligation [1, 2, 3, 4]. The target portosystemic pressure gradient (PPG) for managing acute haemorrhage or preventing recurrence is typically < 12 mmHg, which is largely influenced by shunt diameter [3, 4, 5]. Over‐shunting can severely diminish hepatic perfusion, increasing the risk of post‐TIPS hepatic encephalopathy (HE) and liver failure. Historical studies have indicated that HE incidence remains high, ranging from 22% to 53% at 1 year, even with the use of 10‐mm covered stents in TIPS since 2004 [6, 7, 8]. Conversely, the use of 8‐mm stents may offer advantages in reducing HE and improving survival compared with 10‐mm stents while also outperforming medical therapy in preventing rebleeding from oesophageal varices [9, 10, 11].

Despite these findings, consensus on optimal stent diameter selection has been lacking until recent international guidelines recommend initiating 8‐mm TIPS placement with an 8‐mm diameter stent [4, 5]. However, in regions where body surface area is smaller and hepatitis B virus (HBV) infection predominates as the cause of cirrhosis, initiating TIPS with an 8‐mm stent does not significantly reduce HE and may increase hepatic insufficiency by diverting excessive portal blood flow away from the liver, thereby increasing liver failure rates (14%–20%) [9, 10, 11, 12, 13]. A recent nonrandomized prospective study showed that HE developed in significantly fewer patients with TIPS stents dilated to diameters as low as 6–7 mm (27%) compared with those receiving nominal diameters (8–10 mm; 54%), without differences in recurrent variceal bleeding or ascites and without stent thrombosis [14]. Therefore, a 7‐mm stent diameter may be proposed as an initial option for TIPS placement in patients at risk for post‐TIPS HE and liver failure [8, 9, 10]. Further head‐to‐head studies investigating the relationship between stent diameter and shunt function are warranted in patients susceptible to post‐TIPS HE and liver failure, particularly among patients with failed secondary prophylactic therapy and relatively small liver, as outcomes with different stent diameters requires more robust data. However, for TIPS sizing at diameters < 8 mm, there remains a clear lack of randomized controlled trials (RCT) in patients with failed secondary prophylactic therapy and relatively small liver. Consequently, we conducted this RCT to compare the clinical effectiveness of 7‐mm and 8‐mm TIPS for preventing variceal rebleeding in a cohort of Chinese cirrhotic patients with a relatively small liver.

## 2 Methods

### 2.1 Study Design and Participants

This was an RCT conducted across seven tertiary hospitals, aiming to compare 7‐mm covered TIPS with 8‐mm covered TIPS for preventing variceal rebleeding in patients with cirrhosis and relatively small livers. TIPS was performed immediately after randomization, preferably within 1–2 days. All patients were systematically assessed, and precipitating factors for overt HE (OHE) were documented by investigators who had received uniform standardized training at each site. None of the patients received any pharmacological treatment to prevent the occurrence of OHE. The present report corresponds to the prespecified primary analysis of that trial; no major protocol amendments affecting the primary endpoints or key eligibility criteria were made.

Patients were eligible for the study if they presented with variceal haemorrhage and failed standard secondary prophylactic therapy [1, 3, 4, 5]. After stabilization and successful endoscopic haemostasis, patients were randomly assigned to receive TIPS placement with either 7‐mm or 8‐mm covered stents. The patients' inclusion criteria were as follows: (1) age 18–75 years; (2) liver volume < 1500 cm^3^ [15]; (3) Child‐Pugh score ≤ 13, aspartate aminotransferase (ALT) and alanine aminotransferase (AST) < 5 × the upper limit of normal, alkaline phosphatase < 4 × the upper limit of normal, international normalized ratio (INR) < 2.3, serum creatinine < 1.5 × the upper limit of normal; (4) provision of written informed consent. The following were the exclusion criteria: (1) hepatocellular carcinoma or other malignancies; (2) severe active infection (> Grade II, NCI‐CTCAE 3.0); (3) hypertension with systolic pressure > 150 mmHg or diastolic pressure > 90 mmHg despite proper management; (4) a history of severe or refractory HE; (5) significant heart failure (New York Heart Association class III and IV); (6) absolute contraindications to TIPS; (7) portal vein thrombosis; (8) prior portosystemic shunt or TIPS; (9) sepsis and/or multiorgan failure.

### 2.2 Randomization

This study employed a central random method, and each participating center competed for enrollment. After the investigator received written informed consent, the randomization sequence was computer‐generated by a clinical research coordinator independent of data collection and analysis, using a web‐based allocation system (http://openrct.fmmu.edu.cn) and Pocock and Simon's minimization method at a ratio of 1:1 [16] Randomization was balanced according to study center, sex (male and female) and Child‐Pugh class (A, B, C). The patients were assigned to receive either a 7‐mm or 8‐mm covered stent by contacting the coordinating center after the completion of their preoperative evaluation. Treatment codes were maintained at the coordinating center in sealed, consecutively numbered, opaque envelopes prepared by a research nurse not involved in the study. Investigators performing the TIPS procedure were not blinded to treatment allocation and were not involved in the post‐TIPS management, data collection and analysis. Patients, caregivers, follow‐up investigators (involved in collecting data and assessing endpoints), and the statistician were blinded to treatment assignment.

### 2.3 TIPS Procedures

All procedures were conducted within 48 h after randomization and were performed by experienced interventional radiologists who have performed at least 100 independent procedures. TIPS procedures were performed using a standard technique, as previously described [17, 18]. Specifically, after successful puncture, the 7‐mm stent group uniformly used 7‐mm balloons for predilation, whereas the 8‐mm group uniformly used 8‐mm balloons for predilation. A Fluency stent with a nominal diameter of 7‐mm or 8‐mm was subsequently implanted according to the randomization scheme. The optimal stent position was defined as the cephalic end extending to the hepatocaval junction and the caudal end kept parallel to the vascular wall of the main portal vein. During the procedure, the portosystemic pressure gradient (PPG) was measured preoperatively (baseline PPG) and immediately after stent placement (immediate PPG). PPG values at each defined time point were obtained by averaging the results of three repeated measurements by using a Mindray monitor (Mindray Inc., Shenzhen, China). All patients were following the energy intake of 35–40 kcal/kg body weight/day and protein intake of 1.2–1.5 g/kg body weight/day, without receiving lactulose and/or rifaximin treatment until OHE occurred. No anticoagulants were used, as they are not recommended in TIPS guidelines and may influence the primary endpoint.

### 2.4 Endpoints and Definitions

The primary endpoint of the study was the incidence of OHE after TIPS construction. Covert HE was defined as a psychometric HE score (PHES) ≤ −4 [19]. OHE was defined as HE grade ≥ II according to the modified West–Haven classification [19]. An episode of OHE was defined as the presence of asterixis, inappropriate behaviour, disorientation or HE bouts occurring at an interval of > 6 months [19]. Spontaneous OHE was defined as at least one episode of OHE without any identifiable precipitants [19]. Refractory HE was defined as the persistence of altered mental status despite protein restriction and optimal treatment with lactulose and/or nonabsorbable antibiotics [19]. The secondary endpoints included the following: (1) all‐cause rebleeding, defined as the recurrence of melena or hematemesis after stool colour normalization that resulted in hospitalization, blood transfusion or a haemoglobin decrease of at least 3 g [2, 4, 5]; (2) shunt dysfunction (shunt stenosis or occlusion), identified by ultrasound findings (occlusion, stenosis, > 50% reduction in portal blood flow velocity or velocity < 28 cm/s or reversal of intrahepatic portal flow) combined with high‐risk varices on endoscopy and confirmed by angiography. Shunt dysfunction was defined as ≥ 50% stenosis, complete occlusion, PPG > 12 mmHg or a 25% increase in PPG when post‐TIPS PPG > 12 mmHg [3, 4, 5]; (3) survival, with deaths from any cause recorded. Deaths occurring within 6 weeks after bleeding were classified as bleeding‐related, and deaths within 30 days of TIPS placement as early mortality; (4) hepatic myelopathy, defined as recommended in the recent and most‐updated guidelines [19]; (5) post‐TIPS ascites, defined as the need for performing at least one evacuation of ascitic fluid with paracentesis [3, 4, 5]; and (6) small liver, defined as patients with cirrhosis and liver volumes < 1500 cm^3^ [15].

### 2.5 Follow‐Up

Scheduled follow‐up visits were conducted at 1, 3 and 6 months and every 6 months thereafter, or whenever patients experienced clinical relapse or any other event requiring hospital admission. Assessments include clinical evaluation, physical examination, shunt patency, HE status and laboratory tests. Telephonic follow‐ups were conducted between visits to record patient’ status and details of the clinical events. The patients were followed until death or until the last enrolled patient completed 2 years of follow‐up.

Shunt revision was considered upon detection of shunt dysfunction. Patients with severe HE (> Grade II) or recurrent/persistent HE (≥ 3 OHE episodes within 6 months or prolonged behavioural alterations with recurrent OHE) were considered for shunt reduction [3, 4, 5].

### 2.6 Sample Size Calculation

The sample size was calculated based on the cumulative OHE rates of 43.0% (23/53) in the 8‐mm covered stent group and 20.0% (7/35) in the 7‐mm covered stents group [14]. Using PASS 2011 (NCSS, Kaysville, UT) statistical software, 92 patients per group, including a 10% dropout rate, were required to achieve 80% power at a two‐sided *α* level of 0.05.

### 2.7 Imaging‐Based Liver Volumetry

Liver volumetry was performed using contrast‐enhanced CT with commercial software (IntelliSpace Portal, Philips, Best, the Netherlands). Liver volume was calculated using a semi‐automatic 9‐lobe image segmentation algorithm [20]. The output variable was liver volume, which was reported in cm^3^.

### 2.8 Statistical Analyses

The patients were censored at loss to follow‐up, last outpatient visit before study closure or therapy switching. Continuous variables were analysed after normality testing: normally distributed data were compared using Student's *t*‐test and reported as mean ± standard deviation (Mean ± SD), while non‐normally distributed data were compared using the Mann–Whitney *U* test and presented as median (interquartile range, M (P25‐P75)). Categorical variables were analysed with the chi‐square test (*χ* ^2^‐test) or Fisher's exact test and reported as frequency and percentage (*n*, %). Time‐to‐event outcomes were analysed using Kaplan–Meier curves and log‐rank tests. Cox regression models identified independent predictors, incorporating follow‐up events as time‐dependent variables. Variables with *p* < 0.05 in univariate analysis were entered into multivariable models. Logistic regression was used for binary dependents. Post hoc competing risk survival analyses were conducted, with death and OLT considered as competing events using Grey's test to compare the cumulative incidence function (CIF), and the Fine‐Grey proportional subdistributed risk model was used to estimate the subdistributed risk ratio (sHR). For groups with zero events, Firth's corrected likelihood ratio tests were used, with 95% hazard ratios reported via likelihood confidence limits. All analyses were performed using IBM SPSS Statistics v.25 (IBM Corporation, Armonk, NY, USA), with two‐sided *p* < 0.05 as the universal significance threshold.

## 3 Results

### 3.1 Patients, Recruitment and Follow‐Up

**FIGURE 1:**
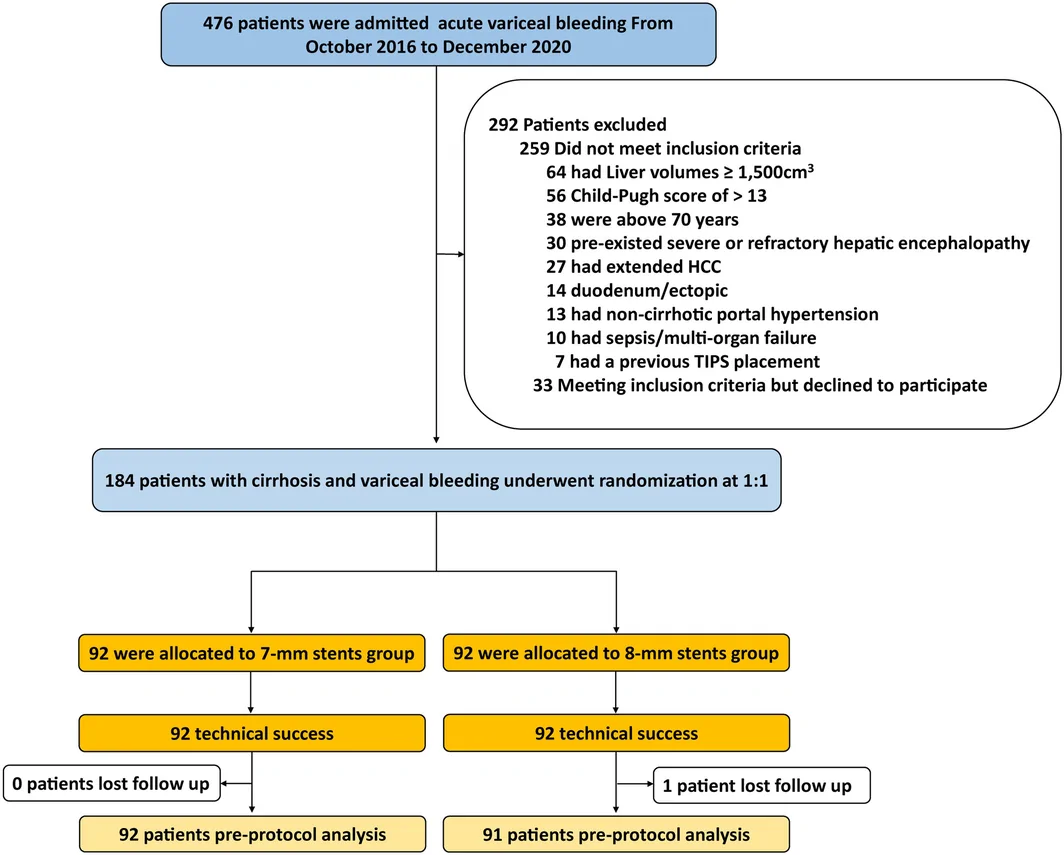
A flowchart of patient enrollment.

The flowchart detailing patient enrollment is presented in Figure 1. Recruitment occurred from October 2016 to December 2020. A total of 184 consecutive cirrhotic patients who failed secondary prophylaxis failed for variceal rebleeding were randomly assigned to receive TIPS either with a 7‐mm stent (92 patients) or an 8‐mm stent (92 patients). The predominant etiologies of liver cirrhosis were mainly HBV (56.0%) and alcohol‐related liver disease (20.7%). Baseline characteristics were comparable between groups (Table 1). The median follow‐up duration was approximately 25.5 months (IQR 24.8–27.1) for the TIPS with a 7‐mm group and 26.3 months (IQR 25.2–27.2) for the TIPS with an 8‐mm group (*p* = 0.406, Table 2). One patient from the TIPS with an 8‐mm group was lost to follow‐up after 18 months.

**TABLE 1:** Baseline characteristics of enrolled patients.

Variables	7‐mm group (*n* = 92)	8‐mm group (*n* = 92)	*p*
Age (years), median (IQR)	57 (44–64)	52 (45–61)	0.07
Male/Female, *n* (%)	59 (64.1)/33 (35.9)	63 (68.5)/29 (31.5)	0.53
Aetiology, *n* (%)			
Hepatic B virus	51 (55.4)	52 (56.5)	0.59
Hepatic C virus	15 (16.3)	17 (18.5)	
Alcoholic	20 (21.7)	18 (19.6)	
Autoimmune liver disease	6 (6.7)	5 (5.4)	
Platelets (×10^9^/L), median (IQR)	62 (55–81)	63 (52–89)	0.46
Haemoglobin (g/L), median (IQR)	83 (75–99)	82 (71–101)	0.28
Prothrombin time (S), median (IQR)	14.1 (12.9–16.4)	14.1 (12.9–16.4)	0.76
INR, median (IQR)	1.3 (1.2–1.4)	1.3 (1.2–1.5)	0.54
Total bilirubin (μmol/L), median (IQR)	26.6 (22.4–33.7)	27.5 (18.5–39.7)	0.81
ALT (U/L), median (IQR)	20.5 (15.0–32.0)	23.0 (17.3–34.0)	0.14
AST (U/L), median (IQR)	30.0 (22.0–41.8)	30.0 (24.0–40.8)	0.93
ALP (U/L), median (IQR)	90.5 (70.3–112.0)	81.0 (69.0–118.0)	0.41
Albumin (g/dL), median (IQR)	35.8 (34.2–39.8)	35.7 (32.6–39.1)	0.72
Blood ammonia (μmol/L), median (IQR)	49.0 (31.1–60.5)	40.6 (27.5–56.8)	0.14
Na^+^ (mmol/L)	141 (139–143)	140 (138–142)	0.12
K^+^ (mmol/L)	4.0 (3.7–4.4)	4.1 (3.7–4.3)	0.70
Creatinine (μmol/L) median (IQR)	62.5 (50.0–73.8)	65.0 (57.3–73.0)	0.15
BUN (mg/dL), median (IQR)	5.2 (4.1–6.5)	5.2 (4.2–6.2)	0.92
Liver volumes (Median, IQR) (cm^3^)	956.4 (861.3–1270.8)	963.2 (878.7–1271.4)	0.18
Ascites, *n* (%)			
Absent	42 (45.7)	44 (47.8)	0.51
Present without LVP	45 (48.9)	41 (44.6)	
Persistent present needing LVP	5 (5.4)	7 (7.6)	
Child‐Pugh Score, median (IQR)	8 (7–11)	8 (7–10)	0.89
Child‐Pugh class, *n* (%)			
A	33 (35.9)	28 (30.4)	0.73
B	53 (57.6)	57 (62.0)	
C	6 (6.5)	7 (7.6)	
MELD score	11.2 ± 2.8	10.8 ± 3.2	0.77
MELD‐Na	11.6 ± 3.1	10.6 ± 2.6	0.47
Variceal type, *n* (%)			
EVs/GOV1	65 (70.7)	67 (72.8)	0.58
GOV2/IGV1	27 (29.3)	25 (27.2)	
Standard secondary prophylaxis			
EBL + NSBB, *n* (%)	68 (73.9)	73 (79.4)	0.68
EBL, *n* (%)	15 (16.3)	12 (13.0)	
IHL + NSBB, *n* (%)	9 (9.8)	7 (7.6)	
Pre‐TIPS one episodic of OHE, *n* (%)	8 (8.7)	7 (7.6)	0.97
Pre‐TIPS covert OHE (PHES ≤ −4), *n* (%)	31 (33.6)	29 (31.5)	0.88
Pre‐TIPS PPG (mmHg), median (IQR)	25 (21–29)	25 (21–28)	0.98

Abbreviations: ALP, alkaline phosphatase; ALT, Alanine aminotransferase; AST, Aspartate aminotransferase; BUN, blood urea nitrogen; EBL, Endoscopic band ligation; EV, oesophageal varix; GOV1, type 1 gastroesophageal varix; GOV2, type 2 gastroesophageal varix; IGV1, type 1 isolated gastric varix; IHL, Injection histoacryl‐lipiodol; IQR, interquartile range; LVP, large‐volume paracentesis; MELD, model for end‐stage liver disease; NSBB, non‐selective beta‐blocker; PPG, portosystemic pressure gradient; TIPS, transjugular intrahepatic portosystemic shunt.

**TABLE 2:** Summary of outcome measurements 2 years after TIPS in the ITT population.

Outcome	7 mm group (*n* = 92)	8 mm group (*n* = 92)	ARR (95% CI)	*p*
Follow‐up (months), median (IQR)	25.5 (24.8–27.1)	26.3 (25.2–27.2)		0.40
Post‐TIPS PPG (mmHg), median (IQR)	10.6 (8.2–13.5)	9.4 (8.3–13.8)		0.52
Post‐TIPS PPG < 12 mmHg, *n* (%)	71 (77.2)	73 (79.3)		0.74
Liver volume (median, IQR) (cm^3^)	956.4 (861.3–1273.8)	963.2 (878.7–1272.4)		0.18
≤ 959 cm^3^	51 (55.4)	41 (44.6)		0.78
> 959 cm^3^	41 (44.6)	51 (55.4)		
Post‐TIPS covert HE	26 (28.3)	29 (31.5)		0.63
Primary outcome				
First episode of overt HE	19 (20.7)	33 (35.9)	−15.2 (−27.6 to −1.2)	0.02
Frequency of overt HE				
One episode	15 (16.3)	21 (22.8)	−6.5 (−17.0 to 5.0)	0.27
More than one episode	4 (4.3)	12 (13.0)	−8.7 (−17.5 to 4.0)	0.04
HE grades				< 0.001
None	53 (57.6)	27 (29.3)		0.001
1	20 (21.7)	32 (34.8)		0.049
2	11 (12.0)	22 (23.9)		
3	7 (7.6)	9 (9.8)		
4	1 (1.1)	2 (2.2)		
Severe HE (Grade 3/4)	8 (8.7)	11 (12.0)	−3.3 (−12.5 to 5.9)	0.47
Spontaneous overt HE	15 (16.3)	29 (31.5)	−15.2 (−27.0 to −2.9)	0.02
Precipitating overt HE	4 (4.3)	5 (5.4)	−1.0 (−7.8 to 5.4)	0.70
Precipitating events				
Higher protein intake	2 (2.2)	2 (2.2)		0.80
Constipation	1 (1.1)	1 (1.1)		
Variceal bleeding	1 (1.1)	0 (0)		
Dehydration	0 (0)	1 (1.1)		
Infections	0 (0)	1 (1.1)		
Refractory HE	0 (0)	1 (1.1)	−1.1 (−5.9 to 3.0)	0.32
Secondary outcome				
Shunt dysfunction	8 (8.7)	8 (8.7)	0 (−8.6 to 8.6)	1.00
All cause rebleeding	10 (10.9)	9 (9.8)	1.1 (−8.1 to 10.3)	0.81
Variceal rebleeding	8 (8.7)	7 (7.6)	1.1 (−7.3 to 9.6)	0.79
PHG	2 (2.2)	2 (2.2)	0 (−5.6 to 5.6)	1.00
Hepatic myelopathy	0 (0.0)	1 (1.1)	−1.1 (−5.9 to 3.0)	0.32
Death	8 (8.7)	11 (12.0)	−2.7 (−10.5 to 4.6)	0.58
Liver failure	0 (0.0)	3 (3.3)		
Sepsis/pneumonia	2 (2.2)	2 (2.2)		
Rebleeding	3 (3.3)	2 (2.2)		
Other	2 (2.2)	3 (2.2)		
Liver transplantation	1 (1.1)	1 (1.1)	0 (−5 to 5)	1.00
OLT‐free survival rates	84 (91.3)	81 (88.0)	1.1 (−7.6 to 9.6)	0.82

Abbreviations: ARR, absolute risk reduction; HE, hepatic encephalopathy; ITT, intention to treat; OLT, orthotopic liver transplantation; PHG, portal hypertension gastropathy; TIPS, transjugular intrahepatic portosystemic shunt.

### 3.2 TIPS Procedure and Hemodynamic Success

TIPS procedures were successfully performed for all participants. To optimize stent positioning, additional stents were implanted in 2 patients in the 7‐mm group and 1 patient in the 8‐mm group. The PPG decreased significantly immediately following stent placement in both arms, without an inter‐group difference (from 25 mmHg (IQR 21–29) to 10.6 mmHg (IQR 8–14) vs. 25 mmHg (IQR 21–28) to 9.4 mmHg (IQR 8–13), *p* = 0.621; Table 2). Among the 184 patients, 71 patients in the 7‐mm group and 73 in the 8‐mm group achieved a PPG < 12 mmHg (77.2% vs. 79.3%, *p* = 0.743; Table 2). The remaining 21 patients in the 7‐mm group and 19 patients in the 8‐mm group who did not achieve this threshold displayed a mean PPG decrease of 53.4% (IQR 45.5–61.2 mmHg) and 54.1% (IQR 41.5–62.2 mmHg), respectively (Table 2 and Figure S1); all 40 patients achieved a PPG decrease of > 20%. Major procedure‐related complications included intra‐abdominal haemorrhage in 1 patient in the 8‐mm group due to puncture of extrahepatic tissues, while 1 patient in the 7 mm group experienced post‐TIPS infection (1.1% vs. 1.1%, *p* = 1.00); both events were non‐fatal and managed effectively.

### 3.3 Primary Endpoint

**FIGURE 2:**
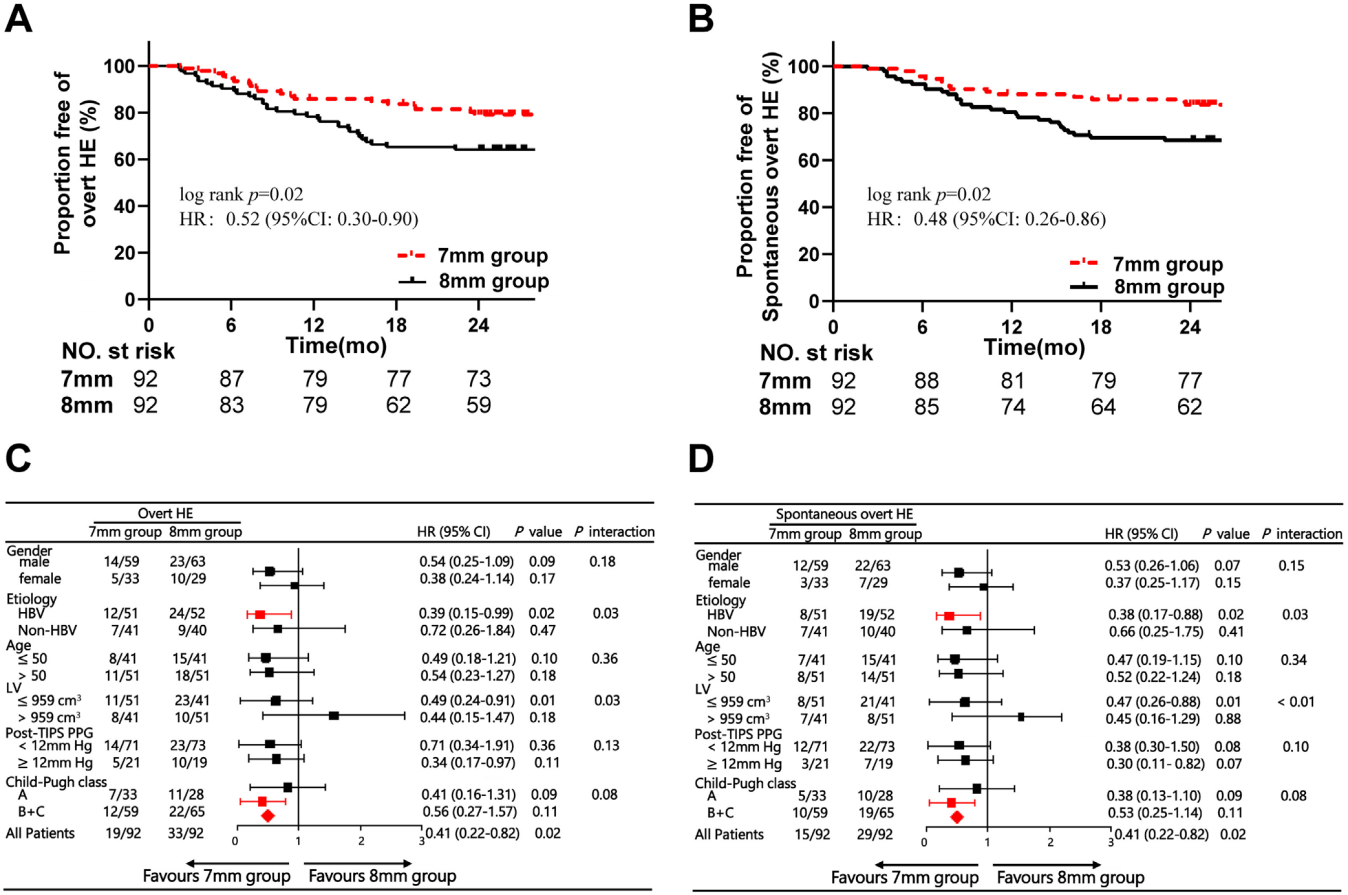
Kaplan–Meier curve of overt hepatic encephalopathy and spontaneous overt hepatic encephalopathy in 184 patients. (A) Proportion free of overt HE in the ITT population. (B) Proportion free of spontaneous overt HE in the ITT population. (C) The incidence of overt HE in randomized 7‐mm vs. 8‐mm arms in subgroups and evaluation of heterogeneity of treatment effect. (D) The incidence of spontaneous overt HE in randomized 7‐mm vs. 8‐mm arms in subgroups and evaluation of heterogeneity of treatment effect. HE, hepatic encephalopathy; ITT, intention to treat; HR, hazard ratio; CI, confidence interval; HBV, hepatitis B virus; LV, liver volume; TIPS, transjugular intrahepatic portosystemic shunt; PPG, portosystemic pressure gradient.

During follow‐up, 19 (20.7%) patients in the 7‐mm group and 33 (35.9%) in the 8‐mm group experienced OHE (*p* = 0.02, Table 2). The 2‐year cumulative incidence rates of OHE were 21.4% in the 7‐mm group vs. 37.2% in the 8‐mm group (HR, 0.52; 95% CI, 0.30–0.90; log‐rank *p* = 0.02; Figure 2A). The incidence of covert HE was also significantly lower in the 7‐mm group (23.6% vs. 38.9%; *p* = 0.049), whereas that of Grade III or IV HE was similar between the two groups. Recurrent HE was significantly less frequent in the 7‐mm group (4.3% vs. 13%; *p* = 0.04; Table 2), whereas episodic HE was similar between the two groups. Overall, the median liver volume was 959 cm^3^ (IQR: 862.4–1270.6): 956.4 cm^3^ (861.3–1273.8) in 7‐mm group and 963.2 cm^3^ (878.7–1272.4) in 8‐mm group. Among 92 patients with liver volumes ≤ 959 cm^3^, OHE was significantly less frequent in the 7‐mm group than in the 8‐mm group (10/51, 19.6% vs. 24/41, 58.5%, *p* < 0.01; Figure 2C). In contrast, among patients with liver volumes > 959 cm^3^, the incidence of OHE did not differ significantly between groups (9/41, 21.9% vs. 9/51, 17.6%, *p* = 0.74; Figure 2C). Among the 44 patients who developed spontaneous OHE, only 20.5% (9/44) had identifiable precipitating factors, likely related to bypass creation and subsequent liver injury following TIPS. More than half of these patients (29/44, 65.9%) belonged to the 8‐mm group. The 2‐year cumulative incidence of spontaneous OHE was consistent with the outcome of OHE and was also significantly lower in the 7‐mm group than in the 8‐mm group (16.3% vs. 32.7%; log‐rank *p* = 0.03, HR: 0.48, 95% CI: 0.26–0.86, Figure 2B), with consistent results in competing risk analysis (sHR = 0.52, 95% CI: 0.26–0.98, Grey's *p* = 0.028, Table S1). Univariable Cox regression identified stent diameter, post‐TIPS PPG, pre‐covert HE, liver volumes and MELD‐Na score were associated with increased OHE risks. In multivariable analysis, only stent diameter, post‐TIPS PPG, pre‐covert HE and MELD‐Na score emerged as independent predictors of OHE (Table 3). The higher post‐TIPS PPG were associated with an increased probability of OHE occurrence, and this relationship was not influenced by liver volumes (Tables S2 and S3). Eight patients in the 7‐mm group and 11 patients in the 8‐mm group developed severe HE (Grade 3/4); all of them were managed without complications. One patient in the 8‐mm group presented with refractory HE and required shunt reduction.

**TABLE 3:** Cox regression analysis for risks of overt HE and OLT‐survival in the ITT population.

Outcomes	Univariate analysis		Multivariate analysis	
	HR (95% CI)	*p*	HR (95% CI)	*p*
Overt HE				
Stent diameter (7 mm vs. 8 mm)	0.45 (0.26–0.79)	0.01	0.52 (0.32–0.88)	0.02
Post TIPS PPG (mmHg)	1.35 (0.98–1.95)	0.01	1.89 (1.21–2.73)	0.02
Pre‐CHE (CHE vs. Non‐CHE)	2.04 (1.02–2.06)	< 0.01	2.06 (1.03–2.69)	0.03
MELD‐Na score	1.08 (1.03–1.13)	< 0.01	1.07 (1.01–1.14)	0.02
LVs (≤ 959cm^3^ vs. > 959cm^3^)	0.96 (0.91–1.00)	0.04		
OLT‐free Survival				
Albumin (g/dL)	0.93 (0.88–0.98)	0.01		
Post TIPS PPG (mmHg)	1.04 (1.02–1.06)	< 0.01	1.15 (0.98–1.24)	0.02
Na^+^ (mmol/L)	0.92 (0.87–0.97)	< 0.01		
MELD‐Na score	1.10 (1.01–1.20)	0.02		
Liver volumes				
≤ 959 cm^3^ vs. > 959 cm^3^	2.06 (1.02–2.06)	< 0.01	2.06 (1.02–1.98)	0.01

Abbreviations: CHE, covert hepatic encephalopathy; CI, confidence interval; HE, hepatic encephalopathy; HR, hazard ratio; MELD, model for end‐stage liver disease; PPG, portosystemic pressure gradient; TIPS, transjugular intrahepatic portosystemic shunt.

Subgroup analysis revealed no significant heterogeneity in treatment effects on OHE and spontaneous OHE across subsets, except for the pre‐covert HE and post‐TIPS PPG (Figure 2C,D).

### 3.4 Secondary Endpoints

**FIGURE 3:**
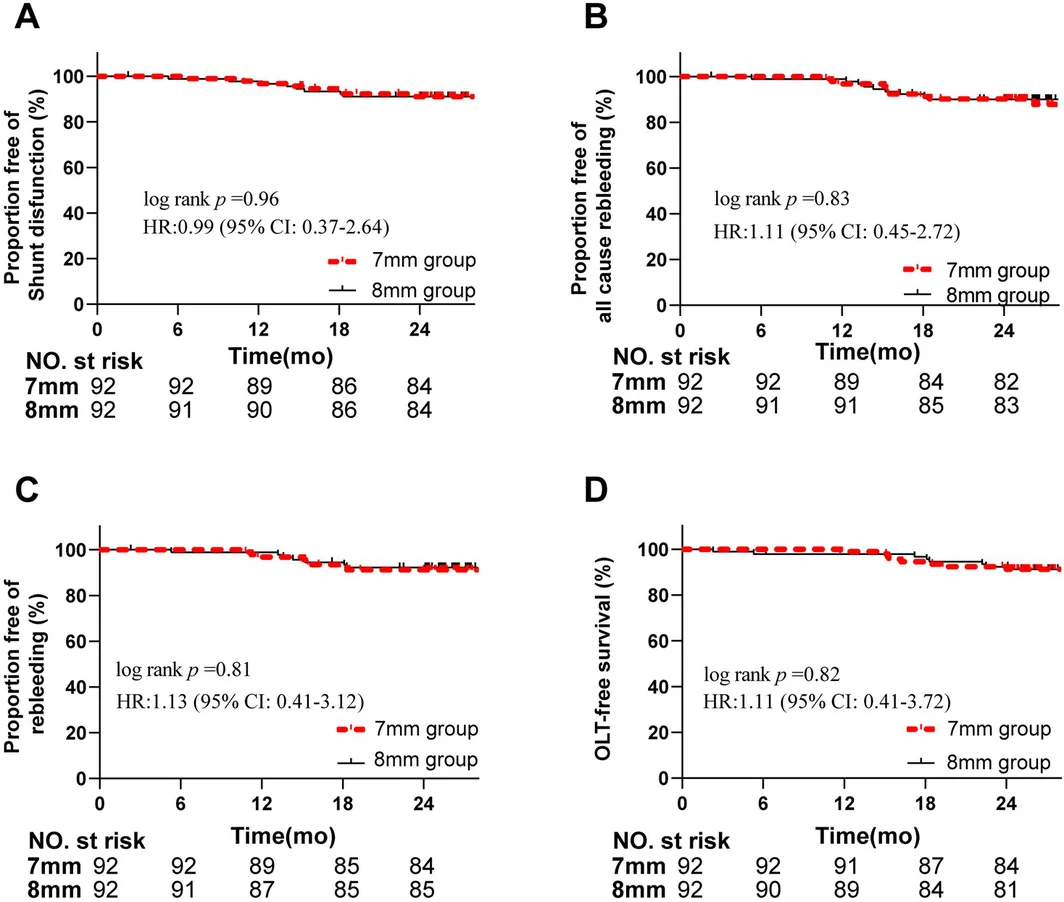
Kaplan–Meier curve of post‐TIPS shunt function and survival in ITT population. (A) Proportion free of shunt dysfunction. (B) Proportion free of all‐cause rebleeding. (C) Proportion free of rebleeding. (D) OLT‐free survival. ITT, intention to treat; HR, hazard ratio; CI, confidence interval; TIPS, transjugular intrahepatic portosystemic shunt; OLT, orthotopic liver transplantation.

A total of 16 patients experienced shunt dysfunction, including 8 from each cohort, accounting for 17 shunt dysfunction events. Among these patients, 8 underwent shunt revision (6 received one revision; the other 2 received two revisions each) via angioplasty, with equal 2‐year rates in both groups (8.7%, 95% CI: 4.5–16.3; *p* = 1.0; Table 2). No significant difference was noted in the cumulative incidence of shunt dysfunction at 2 years for the 7‐mm and 8‐mm stent groups (Log‐rank *p* = 0.96, HR: 0.99, 95% CI: 0.37–2.64; Figure 3A), consistent with post hoc competing risk analyses (sHR = 0.99, 95% CI: 0.32–2.89, Grey's *p* = 0.983, Table S1).

Overall, 10 (10.9%) patients in the 7‐mm group and 9 (9.8%) in the 8‐mm group experienced all‐cause rebleeding (*p* = 0.81; Table 2). All instances required endoscopic evaluations for identifying sources leading to rebleeding occurrences. A total of 13 patients underwent TIPS revisions remaining free thereafter, whereas 6 received medical/endoscopic treatments as primary interventions against rebleeding (5 succumbed due to further bleeding, while 1 patient underwent balloon‐occluded retrograde transvenous obliteration of gastric varices). The 2‐year cumulative incidence rates of all‐cause rebleeding for the 7‐mm and 8‐mm groups were 10.9% [95% CI: 6.0–18.9] and 9.8% [95% CI: 5.2–17.6], respectively (ARR 1.1 [95% CI: −8.1 to 10.3]; HR 0.83 [95% CI: 0.45–2.72]; log‐rank *p* = 0.83; Figure 3B) and competing risk analysis (sHR = 0.79, 95% CI: 0.34–1.78, Grey's *p* = 0.773, Table S1) reported consistent results. The causes of rebleeding are listed in Table 2. Moreover, no significant differences in the 2‐year cumulative incidence rates of variceal rebleeding were observed between both groups (7‐mm group 8.7% [4.5–16.2] vs. 8‐mm group 7.6% [3.7–14.9]; ARR 1.1% [95% CI: 7.3–9.6]; HR 1.13 [95% CI: 0.41–3.12]; log‐rank *p* = 0.81; Figure 3C). One patient in the 8‐mm group experienced hepatic myelopathy, and significant difference was not observed between the two groups in the incidence rate of hepatic myelopathy (Table 2).

**FIGURE 4:**
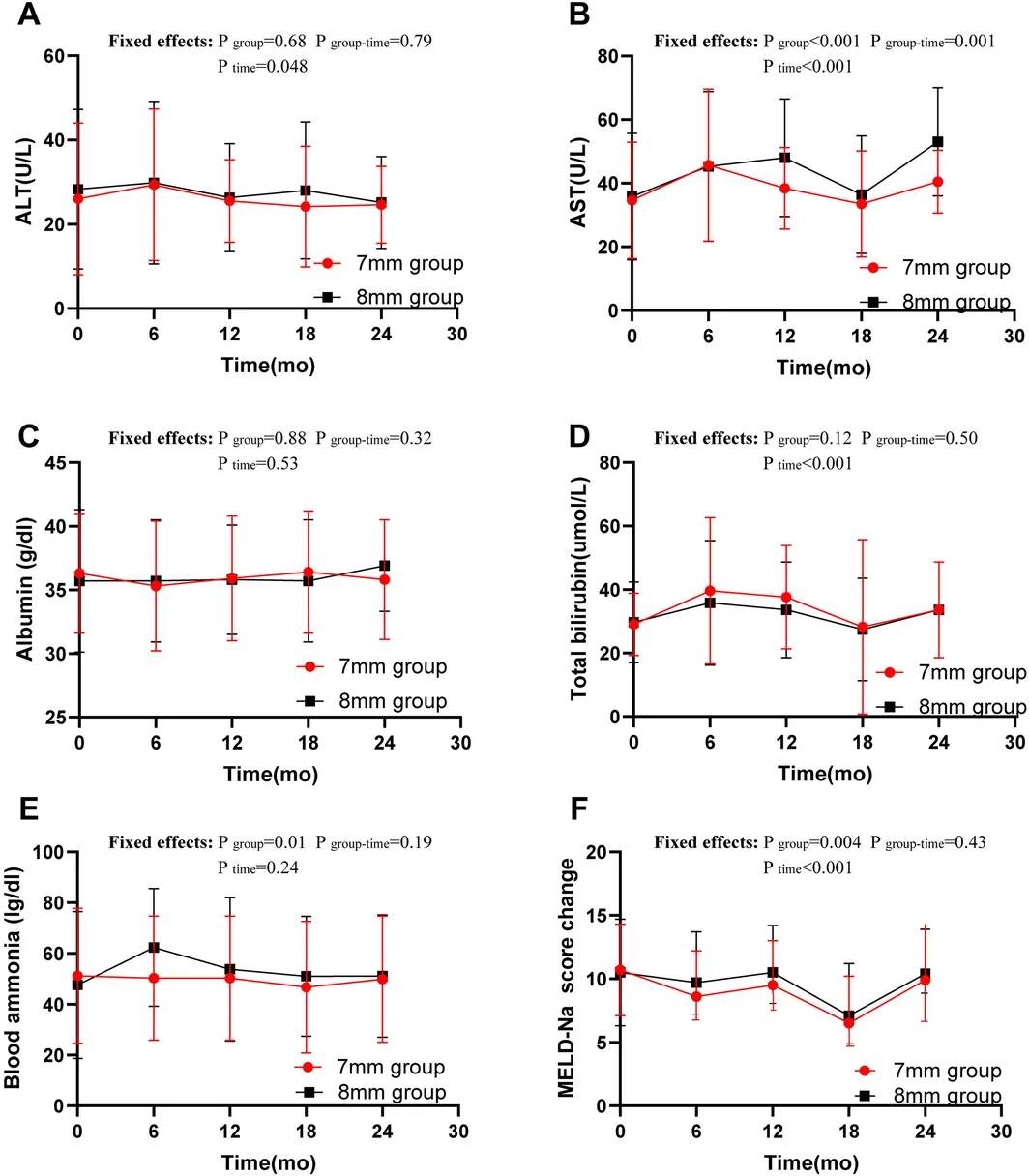
Line chart of ALT, AST, Albumin, total bilirubin and blood ammonia. (A) ALT in the 7‐mm group (red) and 8‐mm group (black). (B) AST in the 7‐mm group (red) and 8‐mm group (black). (C) Albumin in the 7‐mm group (red) and 8‐mm group (black). (D) Total bilirubin in the 7‐mm group (red) and 8‐mm group (black) (E) Blood ammonia in the 7‐mm group (red) and 8‐mm group (black). (F) Line chart of MELD‐Na scores in the 7‐mm group (red) and 8‐mm group (black). ALT, Alanine aminotransferase; AST, Aspartate aminotransferase; MELD, model for end‐stage liver disease.

Laboratory assessments during follow‐up demonstrated the dynamic change in AST levels alongside significantly reduced blood ammonia concentrations; both favoured 7‐mm stents (Figure 4B,E). MELD‐Na scores declined significantly in both groups during six‐month evaluations (*p* < 0001), with more pronounced reductions observed within the 7‐mm cohort (*p* = 0.04, Figure 4F).

**FIGURE 5:**
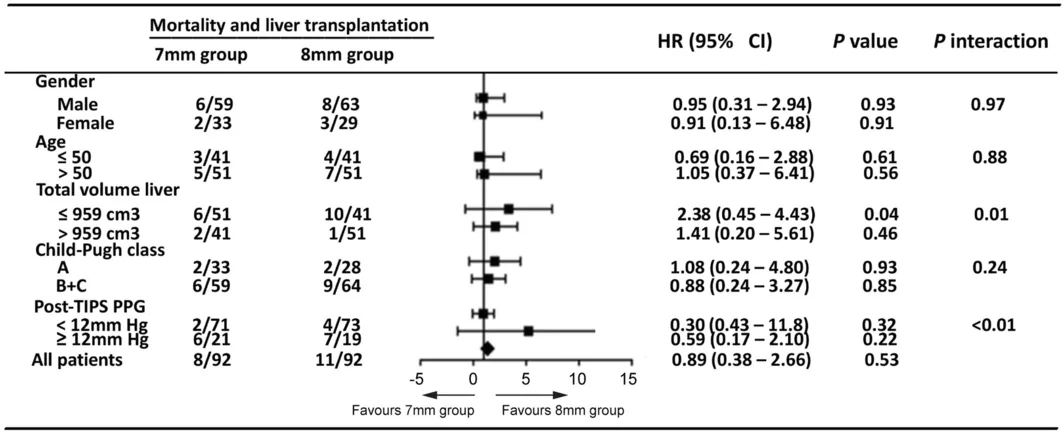
The incidence of mortality and liver transplantation of randomized 7‐mm vs. 8‐mm arms in subgroups and evaluation of heterogeneity of treatment effect. HR, hazard ratio; CI, confidence interval; TIPS, transjugular intrahepatic portosystemic shunt; PPG, portosystemic pressure gradient.

Two patients (one receiving a 7‐mm stent at month 25 and another receiving an 8‐mm stent at month 17) underwent liver transplantation. In total, 19 (8 in the 7‐mm group and 11 in the 8‐mm group) patients succumbed during the study period, and the causes of death are listed in Table 2. The three primary causes of death were liver failure (*n* = 3), sepsis/pneumonia (*n* = 4) and gastrointestinal bleeding (*n* = 5). No significant difference was noted in 2‐year OLT‐free survival rates between the two groups (7‐mm group: 91.3% [95% CI: 3.8–95.5] vs. 8‐mm group: 88.0% [95% CI: 83.8–93.5]; ARR 1.1 [95% CI: −7.6–9.6]; HR 1.00 [95% CI: 0.37–2.66]; log‐rank *p* = 0.82; Figure 3D) and competing risk analysis (sHR = 0.73, 95% CI [0.44–1.28], Grey's *p* = 0.228, Table S1) showed similar results. Univariate analysis identified albumin, post‐TIPS PPG, sodium, MELD‐Na score and liver volumes ≤ 959 cm^3^associated with OLT‐free survival. In multivariable analysis, only post‐TIPS PPG and liver volumes emerged as independent predictors of mortality (Table 3). Subgroup analysis revealed no significant heterogeneity in treatment effects on mortality across subsets, except for post‐PPG ≥ 12 mmHg and liver volumes ≤ 959 cm^3^ (Figure 5).

### 3.5 Other Events

Overall, 31 (33.7%) patients in the 7‐mm group and 25 (27.2%) in the 8‐mm group experienced at least one severe adverse event (*p* = 0.43). No significant differences were noted in the number of patients who experienced a specific adverse event between the groups (Table S4).

## 4 Discussion

This RCT provides the first compelling evidence supporting the feasibility and clinical benefit of 7‐mm covered stents for variceal rebleeding in patients with cirrhosis and relatively small livers. More importantly, this strategy significantly achieved similar shunt function and rebleeding control while significantly reducing the incidence of post‐TIPS OHE. Our study thus suggests a favourable overall outcome of 7‐mm stents and supports the use of 7‐mm TIPS stents among patients with cirrhosis and relatively small livers should be considered as an effective method for preventing variceal rebleeding.

The 7‐mm stent moderately reduces shunt volume by approximately 20% compared with the 8 mm counterpart [21]. Our findings confirmed that moderate shunt volume reduction can effectively lower the translocation of gut‐derived neurotoxins into the systemic circulation—without compromising the key therapeutic target of reducing PPG. Although this study included a cohort of cirrhotic patients with worse liver function and relatively a small liver, the incidence of OHE in the 8‐mm TIPS group was 35.9%, which was significantly higher than that in the 7‐mm TIPS group (20.7%). This result was comparable to the 27.8% in the 6‐mm TIPS, but without increasing rebleeding risk (10.9% vs. 33.3%) [22]. Our results further supported the hypothesis that a larger shunt diameter is associated with an increased risk of post‐TIPS OHE development under similar circumstances. Notably, no patient in the present study received primary prophylactic treatment (rifaximin or lactulose) for OHE. Importantly, in patients with cirrhosis and relatively small liver, our results are the first to confirm that the incidence of post‐TIPS OHE was inversely associated with the diameter of the covered stents, pre‐covert HE, the MELD‐Na score and post‐TIPS PPG. Our findings suggest that 7‐mm stents are suitable for the prophylaxis of variceal rebleeding in patients with failed secondary prophylactic therapy and relatively small liver. This approach was proved as feasible and effective and showed an association with lower post‐TIPS OHE rates.

Shunt patency is the cornerstone for the long‐term prevention of rebleeding. The shunt dysfunction incidence (8.7%) for both groups in the present study was comparable to the 8%–14% for standard covered stents in the landmark study [6, 9]. A multicenter RCT study reported a variceal rebleeding rate of 16.3% 2 years after TIPS placement in patients receiving standard treatment [9]. Additionally, a recent RCT study demonstrated that the all‐cause rebleeding rate after the placement of a 6‐mm TIPS was 33.3% [22], which was higher than the 10.9% rate observed in the present 7‐mm TIPS group. Within the 7‐mm group, the median post‐TIPS PPG was 10.6 mmHg with a median PPG reduction of 57%. Maintaining a PPG level of < 12 mmHg is the primary objective of TIPS to prevent rebleeding. Our study data directly addressed this concern: the rebleeding rates were comparable between the two groups, indicating that 1 mm reduction from 8 mm to 7 mm did not compromise the hemodynamic conditions required for maintaining patency, nor did it increase the risk of rebleeding driven by stent stenosis or thrombosis.

The core principle for selecting the diameter of TIPS stents is to make personalized trade‐offs based on patient‐specific baseline characteristics. The fundamental principle of our comparison between 8 mm and 7 mm stents is to further optimize this balance, thereby reducing the risk of OHE while maintaining the same patency and preventing rebleeding efficacy.

Larger shunts divert more portal blood into the systemic circulation, which reduces liver perfusion [9], potentially contributing to higher mortality. Notably, the mortality rate in our study (8.7%) was significantly lower than the 20.3% reported for standard TIPS with larger shunt diameters [9]. This benefit was particularly evident in patients with Child‐Pugh B/C grade liver function and a smaller liver: the 7 mm stent group exhibited a more pronounced reduction in post‐TIPS OHE incidence without displaying any increase in rebleeding risk. We might explain the potential better overall outcome of 7‐mm stents and support their use to prevent variceal rebleeding in patients with failed secondary prophylactic therapy and relatively small liver as follows: First, the moderate shunt volume reduction can effectively lower the translocation of gut‐derived neurotoxins into the systemic circulation, which is associated with a reduction in the likelihood of OHE development under similar circumstances. Second, the smaller stents may preserve liver function reserve and detoxification capacity by reducing excessive portal blood flow away from the liver, thereby not significantly reducing liver perfusion. Indeed, better liver function reserve in 7‐mm stents supports this idea [3, 4, 5]. Third, smaller stents were advantageous for lowering the incidence of recurrent/persistent HE and severe HE, which was in alignment with the favourable outcome of shunt reduction or occlusion as a therapy for refractory HE. Compared with remedial shunt reduction, smaller stents have the significant advantage of being able to protect against rather than treat OHE. This finding underscores the lack of a survival benefit with 8‐mm stents as the initial TIPS option for the prophylaxis of variceal rebleeding in patients with failed secondary prophylactic therapy and relatively small liver. Our results support the use of 7‐mm stents as the initial option for TIPS placement in patients with cirrhosis and relatively small liver, thereby offering a rationale for extending current practices to optimize treatment outcomes by tailoring stent diameter to patient characteristics.

Despite these promising results, our study has some limitations. First, although this study was conducted across multiple tertiary hospitals, the sample size was relatively small. Nonetheless, the study was sufficiently powered to achieve its primary endpoint. Second, all patients received Fluency stents, not Viatorr, that are now widely used in guideline‐based practice, as the latter were not available in a 7‐mm diameter during the study period. Third, the population is limited to Chinese patients, high HBV prevalence and exclusion of portal vein thrombosis and significant cardiopulmonary comorbidities. Lastly, importing an effect size was derived from non‐randomized studies, which may lead to an overestimation of the actual effect between 7 mm and 8 mm. The conclusions of this study should be further verified through the design of more precise large‐scale studies. Although this limitation must be considered when interpreting the results, quality control was ensured by having all TIPS procedures performed by the same experienced team. Moreover, the use of Fluency stents in both groups minimizes the potential for bias in group comparisons. Despite these limitations, our study provides clinically meaningful data, offering the first comparison of the feasibility and clinical value of 7‐mm versus 8‐mm covered stent grafts for TIPS placement for preventing variceal rebleeding in patients with cirrhosis and relatively small liver.

In conclusion, this study demonstrates that the use of 7‐mm stents achieved a significantly lower risk of OHE, similar rebleeding rates and similar survival rates compared with the use of 8‐mm stents. These findings suggest that the use of 7‐mm stents must be considered to prevent variceal rebleeding in patients with failed secondary prophylactic therapy and relatively small livers who are undergoing TIPS. The use of these stents has the potential to optimize clinical outcomes and influence future clinical practice.

## Author Contributions

All authors were involved with the conception of the manuscript. Yongping Yang, Fuquan Liu and Kan Zhang, Yu Zhang, Quanwei He and Mingming Meng wrote the first draft. Yongping Yang, Yan Chen and Jiangtao Liu provided critical revisions. All authors provided revisions and approved the final manuscript. Yongping Yang is guarantor.

## Funding

This work was supported by the State Key Projects Specialized on Infectious Disease, Chinese Ministry of Science and Technology, 2018ZX10725506; Natural Science Foundation of Hainan Province, 822MS195; Research and Translational Application of Clinical Characteristic Diagnosis and Treatment Technology in the Capital, Z221100007422002.

## Ethics Statement

The study was approved by the institutional review board at each study site, which conformed to the guidelines of the International Conference on Harmonization for Good Clinical Practice. The study has been registered at Clinicaltrials.gov NCT02541825.

## Conflicts of Interest

The authors declare no conflicts of interest.

## Supporting information


**Figure S1:** Comparison of immediate PPG measurements between the 7 mm and 8 mm groups. PPG was non‐difference between groups. PPG, portosystemic pressure gradient.

**Table S1:** Impact of stent size (7 mm vs. 8 mm) on endpoints.

**Table S2:** Impact of PPG and liver volumes on OHE after TIPS.

**Table S3:** The association and interaction between post PPG, LV and post‐TIPS overt HE.

**Table S4:** Post‐TIPS events.

## Data Availability

The data that support the findings of this study are available on request from the corresponding author. The data are not publicly available due to privacy or ethical restrictions.

## References

[1] R. de Franchis , J. Bosch , G. Garcia‐Tsao , et al., “Baveno VII—Renewing Consensus in Portal Hypertension,” Journal of Hepatology 76 (2022): 959–974. 35120736 10.1016/j.jhep.2021.12.022 PMC11090185

[2] G. Garcia‐Tsao , J. G. Abraldes , A. Berzigotti , and J. Bosch , “Portal Hypertensive Bleeding in Cirrhosis: Risk Stratification, Diagnosis, and Management: 2016 Practice Guidance by the American Association for the Study of Liver Diseases,” Hepatology 65 (2017): 310–335. 27786365 10.1002/hep.28906

[3] D. Tripathi , A. J. Stanley , P. C. Hayes , et al., “Transjugular Intrahepatic Portosystemic Stent‐Shunt in the Management of Portal Hypertension,” Gut 69 (2020): 1173–1192. 32114503 10.1136/gutjnl-2019-320221 PMC7306985

[4] E. W. Lee , B. Eghtesad , G. Garcia‐Tsao , et al., “AASLD Practice Guidance on the Use of TIPS, Variceal Embolization, and Retrograde Transvenous Obliteration in the Management of Variceal Hemorrhage,” Hepatology 79 (2024): 224–250. 37390489 10.1097/HEP.0000000000000530

[5] European Association for the Study of the Liver , “EASL Clinical Practice Guidelines on TIPS,” Journal of Hepatology 83 (2025): 177–210. 40180845 10.1016/j.jhep.2025.01.029

[6] S. Angeloni , M. Merli , F. M. Salvatori , et al., “Polytetrafluoroethylene‐Covered Stent Grafts for TIPS Procedure: 1‐Year Patency and Clinical Results,” American Journal of Gastroenterology 99 (2004): 280–285. 15046218 10.1111/j.1572-0241.2004.04056.x

[7] C. Bureau , J. C. Garcia‐Pagan , P. Otal , et al., “Improved Clinical Outcome Using Polytetrafluoroethylene‐Coated Stents for TIPS: Results of a Randomized Study,” Gastroenterology 126 (2004): 469–475. 14762784 10.1053/j.gastro.2003.11.016

[8] I. L. Holster , E. T. T. L. Tjwa , A. Moelker , et al., “Covered Transjugular Intrahepatic Portosystemic Shunt Versus Endoscopic Therapy + β‐Blocker for Prevention of Variceal Rebleeding,” Hepatology 63 (2016): 581–589. 26517576 10.1002/hep.28318

[9] Q. Wang , Y. Lv , M. Bai , et al., “Eight Millimetre Covered TIPS Does Not Compromise Shunt Function but Reduces Hepatic Encephalopathy in Preventing Variceal Rebleeding,” Journal of Hepatology 67 (2017): 508–516. 28506905 10.1016/j.jhep.2017.05.006

[10] J. Trebicka , D. Bastgen , J. Byrtus , et al., “Smaller‐Diameter Covered Transjugular Intrahepatic Portosystemic Shunt Stents Are Associated With Increased Survival,” Clinical Gastroenterology and Hepatology 17 (2019): 2793–2799. 30940552 10.1016/j.cgh.2019.03.042

[11] M. Praktiknjo , J. Abu‐Omar , J. Chang , et al., “Controlled Underdilation Using Novel VIATORR Controlled Expansion Stents Improves Survival After Transjugular Intrahepatic Portosystemic Shunt Implantation,” JHEP Reports 3 (2021): 100264, 10.1016/j.jhepr.2021.100264. 34013182 PMC8113713

[12] C.‐Y. Yu , C.‐H. Lin , and Y.‐H. Yang , “Human Body Surface Area Database and Estimation Formula,” Burns 36 (2010): 616–629. 19900761 10.1016/j.burns.2009.05.013

[13] G. Han , X. Qi , C. He , et al., “Transjugular Intrahepatic Portosystemic Shunt for Portal Vein Thrombosis With Symptomatic Portal Hypertension in Liver Cirrhosis,” Journal of Hepatology 54 (2011): 78–88. 20932597 10.1016/j.jhep.2010.06.029

[14] F. Schepis , F. Vizzutti , G. Garcia‐Tsao , et al., “Under‐Dilated TIPS Associate With Efficacy and Reduced Encephalopathy in a Prospective, Non‐Randomized Study of Patients With Cirrhosis,” Clinical Gastroenterology and Hepatology 16 (2018): 1153–1162. 29378312 10.1016/j.cgh.2018.01.029

[15] B. Gil‐Alzugaray , A. Chopitea , M. Iñarrairaegui , et al., “Prognostic Factors and Prevention of Radioembolization‐Induced Liver Disease,” Hepatology 57 (2013): 1078–1087. 23225191 10.1002/hep.26191

[16] H. W. Cai , J. L. Xia , D. H. Gao , and X. M. Cao , “Implementation and Experience of a Web‐Based Allocation System With Pocock and Simon's Minimization Methods,” Contemporary Clinical Trials 31 (2010): 510–513. 20674777 10.1016/j.cct.2010.07.009

[17] Y. Chen , X. Ma , X. Zhang , et al., “Prevention of Variceal Rebleeding in Cirrhotic Patients With Advanced Hepatocellular Carcinoma Receiving Molecularly Targeted Therapy: A Randomized Pilot Study of Transjugular Intrahepatic Portosystemic Shunt Versus Endoscopic Plus β‐Blocker,” Hepatology International 16 (2022): 1379–1389. 36255564 10.1007/s12072-022-10388-7

[18] Y. Zhang , F.‐Q. Liu , Z.‐D. Yue , et al., “Safety and Efficacy of Transfemoral Intrahepatic Portosystemic Shunt for Portal Hypertension: A Single‐Center Retrospective Study,” World Journal of Clinical Cases 7 (2019): 1410–1420. 31363469 10.12998/wjcc.v7.i12.1410 PMC6656674

[19] H. Vilstrup , P. Amodio , J. Bajaj , et al., “Hepatic Encephalopathy in Chronic Liver Disease: 2014 Practice Guideline by the American Association for the Study of Liver Diseases and the European Association for the Study of the Liver,” Hepatology 60 (2014): 715–735. 25042402 10.1002/hep.27210

[20] T. Zahel , M. Wildgruber , R. Ardon , T. Schuster , E. J. Rummeny , and M. Dobritz , “Rapid Assessment of Liver Volumetry by a Novel Automated Segmentation Algorithm,” Journal of Computer Assisted Tomography 37 (2013): 577–582. 23863535 10.1097/RCT.0b013e31828f0baa

[21] R. N. Srinivasa , R. N. Srinivasa , J. F. B. Chick , A. Hage , and W. A. Saad , “Transjugular Intrahepatic Portosystemic Shunt Reduction Using the GORE VIATORR Controlled Expansion Endoprosthesis: Hemodynamics of Reducing an Established 10‐Mm TIPS to 8‐Mm in Diameter,” Cardiovascular and Interventional Radiology 41 (2018): 518–521. 29038878 10.1007/s00270-017-1807-x

[22] W. Zhang , M. Zhang , J. Xiao , et al., “Efficacy of 6‐Mm and 8‐Mm Transjugular Intrahepatic Portosystemic Shunt for Variceal Bleeding: A Randomized Controlled Trial,” Clinical Gastroenterology and Hepatology 24 (2026): 161–171. 40633893 10.1016/j.cgh.2025.06.023

