# Supplementary material for: A 7‐mm Covered TIPS Reduces Hepatic Encephalopathy Without Increasing Rebleeding in Cirrhotic Patients With Small Liver: A Randomized Study
Source: Liver Int. 2026 Sep 10;46(10):e70875. doi: 10.1111/liv.70875 (PMC13562823; doi:10.1111/liv.70875)
Supplement: Supplementary file 1 [file LIV-46-0-s001.docx]

**Supplementary Table 1 Impact of stent size (7mm vs. 8mm) on endpoints**

| **Post-TIPS overt HE**  **7mm stent CIF 8mm stent CIF** | **sHR (95% CI)** | | **Gray’s** ***P* values** |
| --- | --- | --- | --- |
| **19/92 (20.65%) 33/92 (35.87%)** | | **0.51 (0.28-0.91)** | **0.024** |
| **Post-TIPS spontaneous overt HE**  **7mm stent CIF 8mm stent CIF** | | **HR (95% CI)** | **Gray’s P values** |
| **15/92 (16.30%) 29/92 (31.52%)** | | **0.52 (0.26-0.98)** | **0.028** |
| **Post-TIPS shunt dysfunction**  **7mm stent CIF 8mm stent CIF** | | **HR (95% CI)** | **Gray’s P values** |
| **8/92 (8.67%) 8/92 (8.67%)** | | **0.99 (0.32-2.89)** | **0.983** |
| **Post-TIPS rebleeding**  **7mm stent CIF 8mm stent CIF** | | **sHR (95% CI)** | **Gray’s *P* values** |
| **10/92 (10.87%) 9/92 (9.78%)** | | **0.79 (0.34-1.78)** | **0.773** |
| **Post-TIPS death**  **7mm stent CIF 8mm stent CIF** | | **sHR (95% CI)** | **Gray’s *P* values** |
| **8/92 (8.67%) 11/92 (11.96%)** | | **0.73 (0.44-1.28)** | **0.228** |

**Note:** TIPS, transjugular intrahepatic portosystemic shunt; HE, hepatic encephalopathy; CIF, cumulative incidence functions; CI, confidence interval; sHR, subdistribution hazard ratio.
