# Supplementary material for: A 7‐mm Covered TIPS Reduces Hepatic Encephalopathy Without Increasing Rebleeding in Cirrhotic Patients With Small Liver: A Randomized Study
Source: Liver Int. 2026 Sep 10;46(10):e70875. doi: 10.1111/liv.70875 (PMC13562823; doi:10.1111/liv.70875)
Supplement: Supplementary file 2 [file LIV-46-0-s002.docx]

**Supplementary Table 4 Post-TIPS events**

| Events, n (%) | 7mm group (n = 92) | 8mm group (n= 92) *p* value |
| --- | --- | --- |
| Procedure-related |  |  |
| Intra-abdominal hemorrhage | **0 (0)** | **1 (1.1) 1.000** |
| post-TIPS infection | **1(1.1)** | **0 (0) 1.000** |
| TIPS-related |  |  |
| Post-TIPS ascites | **4(4.3)** | **3(3.3) 1.000** |
|  |  |  |
| Post-TIPS hydrothorax | **2(2.2)** | **1(1.1) 1.000** |
| Post-TIPS peripheral edema | **7(7.6)** | **5(5.4) 0.550** |
| SBP | **1(1.1)** | **1(1.1) 1.000** |
| Hepatorenal syndrome | **3(3.3)** | **2(2.2) 1.000** |
| Hepatic myelopathy | **0 (0)** | **1(1.1) 1.000** |
| non-TIPS adverse events |  |  |
| Peptic ulcer or gastritis | **5(5.4)** | **4(4.3) 1.000** |
|  |  |  |
| Nausea | **4(4.3)** | **3(3.3) 1.000** |
| Fatigue | **5(5.4)** | **6(6.5) 0.756** |

**Note:** Post-TIPS other events were reported from randomization to death, liver transplantation, or when the last randomized patient had been followed up for 2 years. TIPS, transjugular intrahepatic portosystemic shunt.
