# Supplementary material for: A 7‐mm Covered TIPS Reduces Hepatic Encephalopathy Without Increasing Rebleeding in Cirrhotic Patients With Small Liver: A Randomized Study
Source: Liver Int. 2026 Sep 10;46(10):e70875. doi: 10.1111/liv.70875 (PMC13562823; doi:10.1111/liv.70875)
Supplement: Supplementary file 3 [file LIV-46-0-s004.docx]

**Supplementary Table 3 The association and interaction between post PPG, LV and post-TIPS overt HE**

| Outcomes | Univariate logistic analysis | | Multivariate logistic analysis | |
| --- | --- | --- | --- | --- |
|  | **OR (95% CI)** | **p Value** | **OR (95% CI)** | **p Value** |
| 7-mm stents |  |  |  |  |
| LV> 959 cm^3^ | 0.937(0.334-2.665) | 0.904 | 0 (0-75866.128) | 0.341 |
| Post TIPS PPG (mmHg) | 2.080(1.401-3.089) | <0.001 | 2.763 (1.401-5.541) | 0.003 |
| Interaction | ― ― | ― | 1.996 (0.354-11.243) | 0.433 |
| 8-mm stents |  |  |  |  |
| LV> 959 cm^3^ | 0.191(0.076-0.482) | <0.001 | 0 (0-2299520) | 0.374 |
| Post TIPS PPG (mmHg) | 1.646(1.271-2.225) | <0.001 | 6.586(1.955-22.183) | 0.002 |
| Interaction | ― ― | ― | 1.506(0.167-13.576) | 0.715 |

**Note**: HE, hepatic encephalopathy; OR, odds ratio; CI, Confidence interval; TIPS, transjugular intrahepatic portosystemic shun; PPG, portosystemic pressure gradient, LV, liver volumes.
