# Supplementary material for: A 7‐mm Covered TIPS Reduces Hepatic Encephalopathy Without Increasing Rebleeding in Cirrhotic Patients With Small Liver: A Randomized Study
Source: Liver Int. 2026 Sep 10;46(10):e70875. doi: 10.1111/liv.70875 (PMC13562823; doi:10.1111/liv.70875)
Supplement: Supplementary file 4 [file LIV-46-0-s005.docx]

**Supplementary Table 2 Impact of PPG and liver volumes on OHE after TIPS**

| **Variables** | **7mm post-TIPS OHE** | | | **8mm post-TIPS OHE** | | |
| --- | --- | --- | --- | --- | --- | --- |
|  | **Absent**  **(n=73)** | **Present**  **(n=19)** | **P value** | **Absent**  **(n=59)** | **Present**  **(n=33)** | **P value** |
| **Post-PPG≥12mm Hg** | **9** | **12** | **0.001** | **6** | **13** | **0.001** |
| **Post-PP****G<12mm Hg** | **64** | **7** |  | **53** | **20** |  |
| **LV > 959cm^3^** | **34** | **7** | **0.447** | **41** | **10** | **0.001** |
| **LV** **≤ 959cm^3^** | **39** | **12** |  | **18** | **23** |  |

**Note:** PPG, portocaval pressure gradient; HE, hepatic encephalopathy; OHE, overt hepatic encephalopathy; LV, liver volumes.
