# Supplementary material for: A 7‐mm Covered TIPS Reduces Hepatic Encephalopathy Without Increasing Rebleeding in Cirrhotic Patients With Small Liver: A Randomized Study
Source: Liver Int. 2026 Sep 10;46(10):e70875. doi: 10.1111/liv.70875 (PMC13562823; doi:10.1111/liv.70875)
Supplement: Supplementary file 5 [file LIV-46-0-s003.zip › liv70875-sup-0002-FigureS1.docx]

**Supplementary figure Legends**

**Supplementary Figure 1 Comparison of immediate PPG measurements between the 7mm and 8mm groups. PPG was non-difference between groups. PPG, portosystemic pressure gradient.**
